# Increased Dependence of Humans on Ecosystem Services and Biodiversity

**DOI:** 10.1371/journal.pone.0013113

**Published:** 2010-10-01

**Authors:** Zhongwei Guo, Lin Zhang, Yiming Li

**Affiliations:** Key Laboratory of Animal Ecology and Conservation Biology, Institute of Zoology, Chinese Academy of Sciences, Beijing, China; Dalhousie University, Canada

## Abstract

Humans have altered ecosystems more rapidly and extensively than ever, largely to meet rapidly growing demands for resources along with economic development. These demands have been considered important drivers of ecosystem degradation and biodiversity loss. Are humans becoming less dependent on ecosystem services and biodiversity following economic development? Here, we used roundwood production, hydroelectricity generation and tourism investment in 92 biodiversity hotspot and 60 non-hotspot countries as cases to seek the answer. In 1980–2005, annual growth rates of roundwood production, hydroelectricity generation and tourism investment were higher in hotspot countries (5.2, 9.1 and 7.5%) than in non-hotspot countries (3.4, 5.9 and 5.6%), when GDP grew more rapidly in hotspot countries than non-hotspot countries. Annual growth rates of per capita hydropower and per capita tourism investment were higher in hotspot countries (5.3% and 6.1%) than in non-hotspot countries (3.5% and 4.3%); however, the annual growth rate of per capita roundwood production in hotspot countries (1%) was lower than in non-hotspot countries (1.4%). The dependence of humans on cultural services has increased more rapidly than on regulating services, while the dependence on provisioning services has reduced. This pattern is projected to continue during 2005–2020. Our preliminary results show that economic growth has actually made humans more dependent upon ecosystem services and biodiversity. As a consequence, the policies and implementations of both economic development and ecosystems/biodiversity conservation should be formulated and carried out in the context of the increased dependence of humans on ecosystem services along with economic development.

## Introduction

Numerous studies have documented that our Earth is increasingly a human-dominated planet [1]–[4]. Natural ecosystems are under enormous pressure around the world from the growing demands we place on them. Growth in human populations and prosperity translates into increased conversion of natural ecosystems to agricultural, industrial, or residential use, but also into increased demand for ecosystem inputs, such as fresh water, fiber, and soil fertility, as well as increased pressure on the capacity of natural ecosystems to assimilate our waste, including air and water pollution as well as solid waste [5]–[7]. Economic development has posed serious challenges to ecosystems and biodiversity conservation. None of biodiversity hotspots (areas rich in endemic species and threatened by human activities) have more than one-third of their pristine habitat remaining. Historically, they covered 12% of the land's surface, but today their intact habitat covers only 1.4% of the land [8]. The Millennium Ecosystem Assessment (MA) documented the importance of ecosystem services to human well-being and showed that continued supply of these services is threatened by unsustainable anthropogenic activities [9]. Approximately 60% of the ecosystem services examined during the MA are being degraded or used unsustainably, including wood, fresh water, air and water purification, and the regulation of regional and local climate and natural hazards [9]. Over the period of 1990–2005, the world total forest area decreased by 3.1%, while the global GDP increased by about 32%. Humans have actually reduced well-being that they yield from ecosystem services, owing to human-induced changes to components of the Earth's biodiversity and ecosystems along with economic development. Are humans becoming less dependent on ecosystem services and biodiversity?

Relationships between ecosystem services and human well-being are poorly understood [10]. Most research related to ecosystem services focuses on direct drivers, such as land use change or invasive species. Yet, effective management requires more attention to indirect drivers such as demographic, economic, sociopolitical, and cultural factors. Lack of knowledge of trends in human reliance on ecosystem services also posed serious constraint in the MA analysis [10]. Lack of appreciation of humans dependence on natural ecosystems represents but one of a complex of interacting factors responsible for today's array of anthropogenic disruptions of the biosphere. Yet, it clearly represents a major hindrance to the formulation and implementation of policy designed to safeguard earth's life-support systems [11]. Moreover, lack of understanding of the relations between ecosystem services and human well-being traces ultimately to a failure of the scientific community to generate, synthesize, and effectively convey the necessary information to the public. In fact, the benefits provided by natural ecosystems are both widely recognized and poorly understood. Consequently, it is vital to understand the relationships between ecosystem services and human well-being as well as their changes following economic development, including: (*i*) the correlations between human well-being yielded from ecosystem services and economic growth; (*ii*) the dynamics of the dependence of humans on different types of ecosystem services; and (*iii*) the effects of ecosystems and biodiversity on human well-being yielded from ecosystem services.

The MA has provided us with a useful classification of ecosystem services: supporting, provisioning, regulating, and cultural [9]. Supporting services are necessary for the production of all other ecosystem services, including biomass production, soil formation and retention, and provisioning of habitat. Humans usually yield well-being from supporting services through the other three services. Provisioning services are products obtained from ecosystems, for example, roundwood and fruits. Roundwood production is a major way that humans directly yield well-being from natural ecosystems and occurred in over 170 countries and regions during 1980–2005. Regulating services provide well-being for humans by regulating ecosystem processes, for example, climate regulation, flood regulation, etc. For most regulating services, there is a lack of valuations of their provision by natural systems [10]–[13]. Yet, the mechanism of hydroelectricity production benefiting from regulating services (i.e., river water flow regulation and sediment retention) has been understood better than others [14]–[16], and over 160 countries and regions used hydropower in 1980–2005. Cultural services are the nonmaterial benefits people obtain from ecosystems through spiritual enrichment, cognitive development, reflection, recreation, and aesthetic experiences. Tourism is an example of humans yielding well-being from cultural services. In 1988–2005, over 180 countries and regions made investments in tourism, which indicates the demand of humans for cultural services.

In this study, we asked the question: Whether or not humans are becoming less dependent on ecosystem services and biodiversity along with developments? Here, we used roundwood production, hydroelectricity generation and tourism investment as the indicators of human well-being yielded from provisioning, regulating and cultural services, respectively. Using these three indicators, we evaluated the dynamics in the dependence of humans on ecosystem services and the effects of ecosystems and biodiversity on human well-being. To understand the relationship between the dependence of humans on ecosystem services and biodiversity hotspots, we divided 152 countries involved in this study into the two categories: biodiversity hotspot countries and non-hotspot countries, according to whether or not a country is with biodiversity hotspot (Table S1). To quantify the effect of economic development on the dependence of humans on ecosystem services, we analyzed GDP, roundwood production, hydroelectricity generation and tourism investment in 92 biodiversity hotspot and 60 non-hotspot countries, and further in 81 developing and 11 industrialized countries among hotspot countries (Table S2). Moreover, in six representative biodiversity hotspot countries, we investigated the changes in per capita roundwood production (PCRP), per capita hydroelectricity generation (PCHG) and per capita tourism investment (PCTI) to reveal the dependence of humans on different kinds of ecosystem services. Finally, we evaluated effects of ecosystems and biodiversity on roundwood production, hydropower and tourism.

## Results

### Correlations between human well-being yielded from ecosystem services and GDP growth

**Figure 1:**
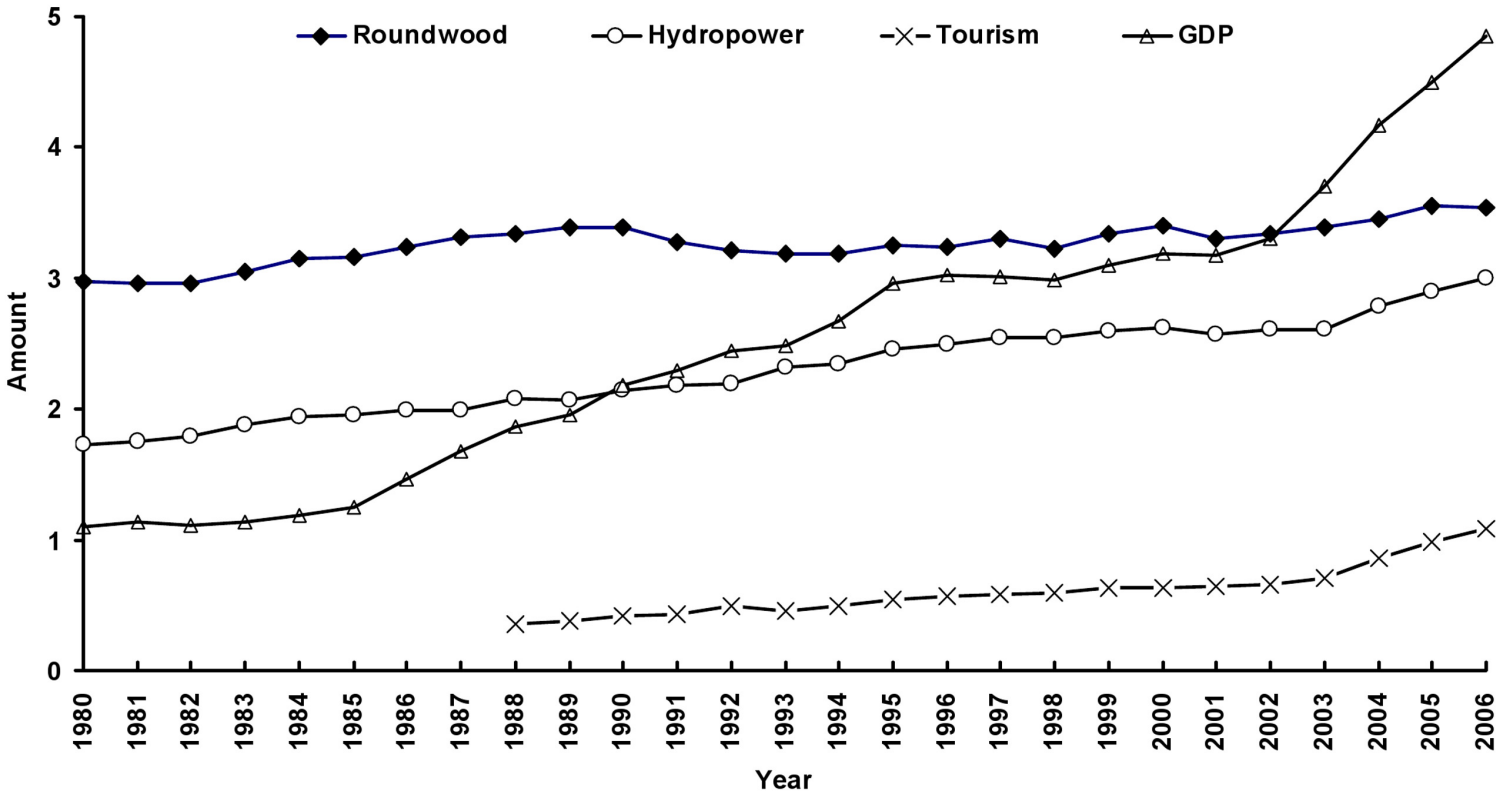
Dynamics of roundwood production, hydropower, tourism investment and GDP in the world between 1980 and 2005.

First, we assessed the correlation between human well-being yielded from ecosystem services and GDP growth at the global scale. Over the period of 1980–2005, the world roundwood production, hydroelectricity generation and tourism investment grew by 17.6, 116.2 and 121.2%, respectively, while GDP increased by 106%. Over the 26 years, 105 countries rose roundwood production, 113 countries increased hydroelectricity generation and 125 countries expanded tourism investment, in the world. The world annual roundwood production and hydroelectricity generation in 1980–2005 and tourism investment in 1988–2005 were positively correlated with annual GDP (*n* = 26, *r* = 0.7489, *p* = 0.0001; *n* = 26, *r* = 0.981, *p*<0.0001; *n* = 18, *r* = 0.976, *p*<0.0001), respectively. Furthermore, by 2005, total roundwood production and hydroelectricity generation since 1980 and tourism investment since 1988 in each country were positively correlated with total GDP in 152 countries (*n* = 152, *r* = 0.663, *p* = 0.008; *n* = 152, *r* = 0.596, *p* = 0.005; *n* = 152, *r* = 0.976, *p* = 0.004). Figure 1 illustrates the dynamics of roundwood production, hydroelectricity generation, tourism investment and GDP in 1980–2005. Moreover, in 2005–2020, the world roundwood production, hydroelectricity generation and tourism investment are projected to increase by about 41%, 44% and 122%, respectively, associated with a projected GDP growth of over 80%.

**Figure 2:**
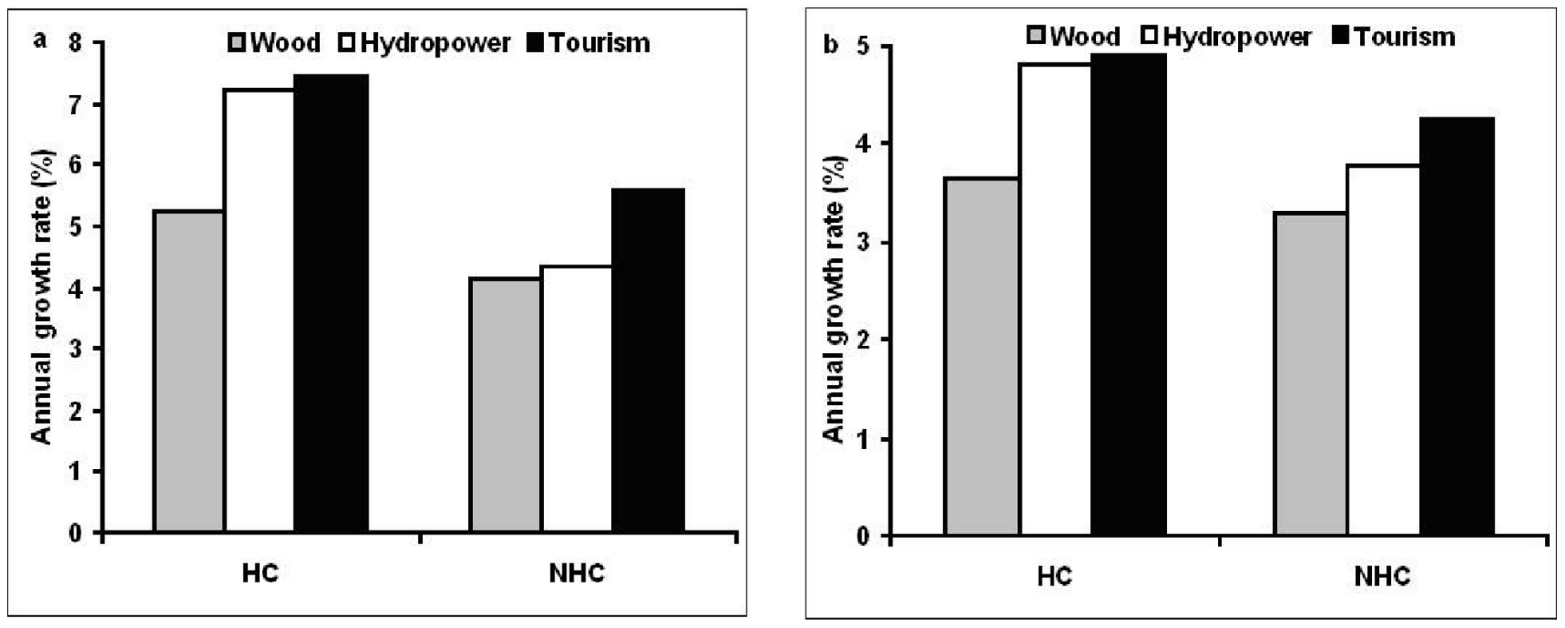
Dynamics of roundwood production, hydropower, tourism investment and GDP in 92 hotspot (HC) and 60 non-hotspot countries (NHC). **a**. Annual growth rates of roundwood production, hydropower, tourism investment and GDP in HC and NHC during 1980–2005. **b**. Projected annual growth rates of roundwood production, hydropower, tourism investment and GDP in HC and NHC during 2005–2020.

**Figure 3:**
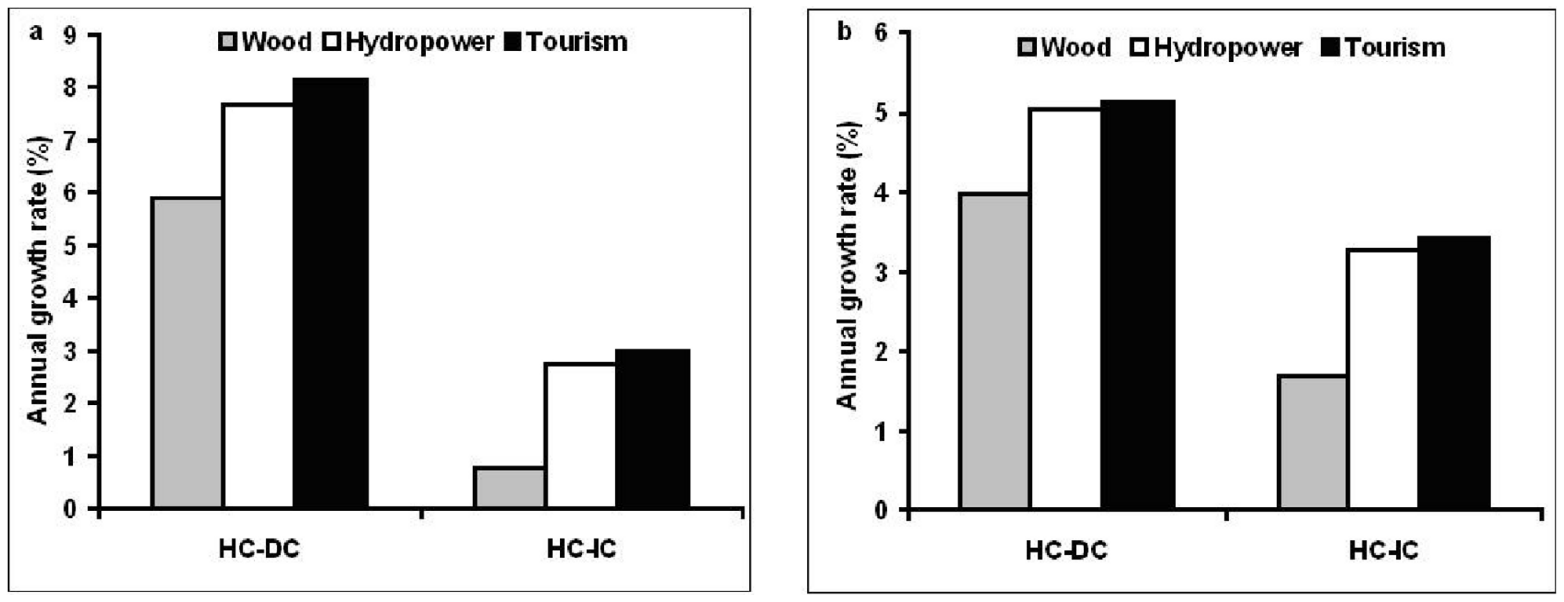
Dynamics of roundwood production, hydropower, tourism investment and GDP in 81 developing hotspot (HC-DC) and 11 industrialized hotspot countries (HC-IC). **a**. Annual growth rates of roundwood production, hydropower, tourism investment and GDP in HC-DC and HC-IC during 1980–2005. **b**. Projected annual growth rates of roundwood production, hydropower, tourism investment and GDP in HC-DC and HC-IC during 2005–2020.

Next, to understand changes of human well-being yielded from ecosystem services and GDP in biodiversity hotspot and non-hotspot countries, respectively, we compared roundwood production, hydroelectricity generation, tourism investment and GDP in the two categories of country. Between 1980 and 2005, in 71% of hotspot countries and 55% of non-hotspot countries, roundwood production, hydroelectricity generation and tourism investment grew following GDP growth. Annual growth rates of roundwood production, hydroelectricity generation and tourism investment were higher in hotspot countries (5.2, 9.1 and 7.5%) than in non-hotspot countries (3.4, 5.9 and 5.6%) (Fig. 2a), when GDP grew more rapidly in hotspot countries than non-hotspot countries. In about 60% of hotspot countries, annual rates of growth in hydroelectricity generation and tourism investment were higher than roundwood growth, while about 30% of non-hotspot countries showed this pattern (Table 1). Over the period 2005–2020, hotspot countries are expected to keep higher growth rates in roundwood production, hydropower and tourism investment (Fig. 2b), along with a higher GDP growth rate, than non-hotspot countries. Moreover, hydroelectricity generation and tourism investment are projected to grow more rapidly than roundwood production in hotspot countries yet during 2005–2020. Among hotspot countries, GDP growth rate was higher in developing countries than in industrialized countries during 1980–2005. In developing hotspot countries, annual growth rates of roundwood production, hydropower and tourism investment were substantially higher than in industrialized hotspot countries between 1980 and 2005 (Fig. 3a). Projected growths in GDP, roundwood production, hydroelectricity generation and tourism investment during 2005–2020 are still higher in developing hotspot countries than in industrialized hotspot countries (Fig. 3b).

**Table 1:** Comparisons of growth rates of roundwood production, hydroelectricity generation and tourism investment.

Time period (years)	Relationship between *RWP*, *HEG* and *TIM*	Hotspot countries		Non-hotspot countries		Developing hotspot countries		Industrialized hotspot countries	
		Number of countries	%	Number of countries	%	Number of countries	%	Number of countries	%
1980–2005	*HEG*(+)>*RWP*(+)§	61	66.3	27	45.0	59	72.8	2	18.2
	*HEG*(+)<*RWP*(+)¦	13	14.1	24	40.0	9	11.1	4	36.4
	*TIM*(+)>*RWP* (+)*	63	68.4	28	46.7	58	71.6	5	45.5
	*TIM*(+)<*RWP* (+)!!	12	13.0	25	41.7	11	13.6	1	9.1
	*HEG*(+)>*TIM*(+)‡	26	28.3	28	46.7	22	27.2	4	36.4
	*HEG* (+)<*TIM* (+)†	54	58.7	24	40.0	49	60.5	5	45.5
2005–2020	*HEG*(+)>*RWP*(+)	60	65.2	24	40.0	55	67.9	5	45.5
	*HEG*(+)<*RWP*(+)	23	25.0	26	43.3	19	23.5	4	36.4
	*TIM*(+)>*RWP* (+)	59	64.1	27	45.0	51	63.0	8	72.7
	*TIM*(+)<*RWP* (+)	20	21.7	26	43.3	19	23.5	1	9.1
	*HEG*(+)>*TIM*(+)	41	44.6	21	32.3	38	46.9	3	27.3
	*HEG*(+)<*TIM* (+)	48	52.2	38	63.3	40	49.4	8	72.7

Abbreviations: *RWP*, annual rate of growth in roundwood production; *HEG*, annual rate of growth in hydroelectricity generation; *TIM*, annual rate of growth in tourism investment. The frequency of hotspot and non-hotspot countries occurring in categories *HEG*(+)>*RWP*(+) and *HEG*(+)<*RWP*(+) differed at *P* = 0.0384 for 1980–2005 and *P* = 0.0293 for 2005–2020. The frequency of hotspot and non-hotspot countries occurring in categories *TIM*(+)>*RWP* (+) and *TIM*(+)<*RWP* (+) differed at *P* = 0.0229 for 1980–2005 and *P* = 0.0284 for 2005–2020.

§ Both *HEG* and *RWP* are positive and the former is greater than the latter.

¦ Both *HEG* and *RWP* are positive and the former is smaller than the latter.

*Both *TIM* and *RWP* are positive and the former is greater than the latter.

!! Both *TIM* and *RWP* are positive and the former is smaller than the latter.

‡ Both *TIM* and *HEG* are positive and the former is greater than the latter.

† Both *TIM* and *HEG* are positive and the former is smaller than the latter.

### Dynamics in the dependence of humans on ecosystem services

Here, we compared the changes of PCRP, PCHG and PCTI in biodiversity hotspot and non-hotspot countries. Between 1980 and 2005, annual growth rates of PCHG and PCTI were higher in hotspot countries (5.3 and 6.1%) than in non-hotspot countries (3.5 and 4.3%). In contrast, the annual growth rate of PCRP was lower in hotspot countries (0.9%) than in non-hotspot countries (1.4%), owing to a higher growth rate of population in hotspot countries. Over the next 16 years, projected growth rates of PCHG and PCTI are still higher in hotspot countries than in non-hotspot countries and PCRP is expected to grow more rapidly in non-hotspot countries than in hotspot countries (Table 2). Between 1980 and 2005, the global annual growth rates of PCHG and PCTI were about 0.6% and 5.6%, on other hand, the global PCRP declined about 0.8% a year. Moreover, during 2005–2020, the global annual growth rates of PCRP, PCHG and PCTI are projected to be 0.7%, 2% and 5.8%, respectively.

**Table 2:** Comparisons of growth rates of per capita roundwood production, per capita hydroelectricity generation and per capita tourism investment.

Time period (years)	Changes in *PCRG*, *PCHG* and *PCTG*	Hotspot countries		Non-hotspot countries		Developing hotspot countries		Industrialized hotspot countries	
		Number of countries	%	Number of countries	%	Number of countries	%	Number of countries	%
1980–2005	*PCRG*>0	32	34.8	33	55.0	25	30.9	7	63.6
	*PCRG*≤0	60	65.2	27	45.0	56	69.1	4	36.4
	*PCHG*>0	72	78.3	39	65.0	66	81.5	6	54.5
	*PCHG*≤0	20	21.7	21	35.0	15	18.5	5	45.5
	*PCTG*>0	79	85.9	41	68.3	70	86.4	9	81.8
	*PCTG*≤0	13	14.1	18	30.0	11	13.6	2	18.2
2005–2020	*PCRG*>0	39	42.4	30	50.0	33	40.7	6	54.5
	*PCRG*≤0	53	57.6	30	50.0	48	59.3	5	45.5
	*PCHG*>0	63	68.5	33	55.0	55	67.9	8	72.7
	*PCHG*≤0	19	20.7	27	45.0	16	19.8	3	27.3
	*PCTG*>0	68	73.9	36	60.0	59	72.8	9	81.8
	*PCTG*≤0	14	15.2	24	40.0	12	14.8	2	18.2

Abbreviations: *PCRG*, annual rate of growth in per capita roundwood production; *PCHG*, annual rate of growth in per capita hydroelectricity generation; *PCTG*, annual rate of growth in per capita tourism investmen**t**. The frequency of hotspot and non-hotspot countries occurring in categories *PCRG*>0 and *PCRG*≤0 at *P* = 0.0206 in 1980–2005. The frequency of hotspot and non-hotspot countries occurring in categories *PCHG*>0 and *PCHG*≤0 differed at *P* = 0.0092 for 1980–2005 and *P* = 0.0006 for 2005–2020. The frequency of hotspot and non-hotspot countries occurring in categories *PCTG*>0 and *PCTG*≤0 differed at *P* = 0.0384 for 1980–2005 and *P* = 0.0293 for 2005–2020.

In the six representative hotspot countries listed in Table 3, PCTI grew more rapidly than PCHG. Between 1980 and 2005, the annual growth rate of PCTI in Brazil, China and Spain was about 10–80% higher than PCHG growth rate and, in Ethiopia, Mexico and New Zealand, the annual rate of growth was even about 3–20 times higher in PCTI than in PCHG. The growth of PCTI is projected to be still higher than the growth of PCHG in the six countries during 2005–2020. PCRP in Brazil, New Zealand and Spain, annual growth rate was about 0.09–1.9% during 1980–2005. In China, Ethiopia and Mexico, PCRP declined at an annual rate of 0.3–0.9% over the period of 1980–2005, owing to a lower annual growth rate of roundwood production (0.8–2.3%). By 2020, PCRP in Brazil, China, New Zealand and Spain is expected to increase by about 1.9–32% since 2005. PCRP in Ethiopia and Mexico will likely decline by 33.8 and 2.4%, respectively, between 2005 and 2020, owing to that roundwood production in Ethiopia is expected to decrease by 0.3% per year and projected roundwood production in Mexico remains at a lower annual growth rate (1.7%). Changes in PCRP, PCHG and PCTI provide the gauge of dynamics of the dependence of humans on ecosystem services.

**Table 3:** Status of six biodiversity hotspot countries in 1980–2005.

	Brazil	China	Ethiopia	Mexico	New Zealand	Spain
Relationship with biodiversity hotspots	Contains a portion of ‘Cerrado’ and ‘Atlantic Forest ’	Contains a portion of ‘Mountains of Southwest China’ and ‘Indo-Burma’	Contains a portion of ‘The Horn of Africa’ and ‘The Eastern Afromontane’	Contains a portion of ‘California Floristic Province’ , ‘Mesoamerica’ and ‘Madrean Pine-Oak Woodlands’	Is identical to ‘New Zealand’	Contains a portion of ‘Mediterranean Basin’
Per capita *GDP* in 2005 (US Dollars)	4271	1698	144	7180	27133	26116
Annual growth rate of roundwood production in 1980–2005 (%)	1.8	0.8	2.3	0.9	2.9	1.1
Annual growth rate of hydropower in 1980–2005 (%)	4.0	8.2	7.5	3.7	1.1	3.4
Annual growth rate of tourism investment in 1980–2005 (%)	3.1	13.3	24.3	8.6	3.2	5.7
Annual growth rate of PCRP in 1980–2005 (%)	0.1	−0.3	−0.3	−0.9	1.9	0.8
Annual growth rate of PCHG in 1980–2005 (%)	2.3	8.3	4.8	1.8	0.1	3.1
Annual growth rate of PCTI in 1980–2005 (%)	2.5	12.2	21.9	7.2	2.0	5.7

We found that biodiversity hotspot countries benefited from ecosystem services more than non-hotspot countries. Over the period of 1980–2005, the mean per country roundwood production, hydroelectricity generation and tourism consumption were about 200%, 60% and 200% higher, respectively, in hotspot countries than in non-hotspot countries. In Brazil, hydropower provided about 92% of total electricity supply and, if wood production in 2005 was the same as in 1980, there would be 30.3% fewer wood fuel supply. In China, had tourism investment remained at the 1988 level, about 87% of tourism demand could not been met in 2005 and, between 1980 and 2005, hydropower consumption per capita grew by 2.3 times. The contribution of tourism industry to Ethiopia's GDP increased from 2.9% to 4% in 1988–2005 and the proportion of hydropower to the total electricity supply in Ethiopia grew from 70% to 98.6% during 1980–2005. By 2005, Mexico added wood fuel supply by 30% and increased tourism consumption by about 3 times since 1980. In New Zealand, hydropower provided near 70% of total electricity supply and roundwood supply increased by over 90%, in 1980–2005. Spain's tourism consumption increased by over 2 times and wood fuel supply grew by 72% over the period of 1980–2005.

### Effects of ecosystems and biodiversity on human well-being yielded from ecosystem services

To understand effects of ecosystems and biodiversity on human well-being yielded from ecosystem services, we analyzed the correlation between roundwood production, hydroelectricity generation and tourism receipts, respectively, and the stocks of ecosystem resources and biodiversity hotspots. Between 1990 and 2005, roundwood production in each country was positively correlated with total forest area in 152 countries (*n* = 152, *r* = 0.555, *p*<0.0001). The mean per country total forest area in 1990–2005 was about 130% higher in hotspot countries than in non-hotspot countries. In the meantime, the mean per country roundwood output was 150% higher in hotspot countries than in non-hotspot countries.

By 2005, total hydroelectricity generation in each country was positively correlated with total forest area since 1990 in 152 countries (*n* = 152, *r* = 0.713, *p* = 0.0003). This result shows the close relation between hydroelectricity generation and natural ecosystems, which is usually neglected. Hotspot countries possess near 80% of the global hydroelectric resources. All of 34 biodiversity hotspots overlap river systems with dams. In Brazil, the ‘Atlantic Forest’ and ‘Cerrado’ hotspots overlap the Paraná, Parnaiba, Tocantins, São Francisco and Uruguay watersheds and 80% of major dams are located in these watersheds. The ‘Mountains of Southwest China’ hotspot overlaps the Huanghe and Yangtze watersheds in China and both watersheds provide over a half of hydropower for China. Hydroelectricity generation in several European countries including Greece, Italy, Portugal, Spain, etc., occurs in ‘the Mediterranean Basin’ hotspot. The ‘Mesoamerica’ hotspot contains over 80% of Mexico's rivers, and all major hydropower plants of Mexico are located at these rivers. The ‘New Zealand’ and ‘Japan’ hotspots cover all river systems in both countries and thus are involved in all hydroelectricity generation. The ‘Eastern Afromontane’ hotspot overlaps the Congo, Nile, Rufiji, Turkana and Zambezi watersheds, where provide about one-forth of hydropower supply in Africa. During 1980–2005, river systems with biodiversity hotspots provided 13–90% of hydropower supply in hotspot countries.

By 2005, tourism receipts were positively correlated with the stock of ecological resources (Forest area+Water body area+Grassland area+Coastline length: F+W+G+C) in the 152 countries (*n* = 152, *r* = 0.606, *p* = 0.0132) since 1988. Our result shows that tourism is close related to natural ecosystems. The average stock of ecological resources in each country (F+W+G+C) is 109.4% higher in hotspot countries than in non-hotspot. The ‘Mediterranean Basin’ hotspot includes the entire Mediterranean shore. Indonesia is covered by the ‘Sundaland’ and ‘Wallacea’ biodiversity hotspots, Madagascar is covered by the ‘Madagascar and the Indian Ocean Islands’ biodiversity hotspot and Thailand is covered by the ‘Indo-Burma’ and ‘Sundaland’ biodiversity hotspots, respectively. The ‘Atlantic Forest’ and ‘Cerrado’ hotspots account for over 60% of Brazil's total vegetation area. The ‘Caribbean Islands’ hotspot comprises more than a dozen countries in the Caribbean region, such as Cuba, Jamaica and Puerto Rico. In China, the three well-known tourist sites, i.e., Three Gorgers, Yunnan and Hainan, are covered by the ‘Mountains of Southwest China’ and ‘Indo-Burma’ hotspots, respectively. Over the period 1988–2005, tourism receipt was 128.4% higher in hotspot countries than in non-hotspot countries.

Between 1990 and 2005, the global economic benefits gaining from roundwood production, hydropower and tourism increased by about 32%; however, the world total forest area decreased by 3.1% over this period. Had total forest area remained at the 1990 level, there would have been 91083 and 23045 thousand hectares more forest area in hotspot and non-hotspot countries (equivalent to 1046.9 and 4.5 thousand hectares additional forest area in each country) in 2005, respectively.

## Discussion

The pursuit of economic growth, indicated by GDP, impels humans to use ecosystem services (such as hydropower). Ecosystem services support economic development and the profits from the use of ecosystem services increase GDP. Thus economic growth relies on ecosystem services. The expansions of roundwood production, hydropower and tourism provide examples of increased dependence of humans on ecosystem services and biodiversity and this increased dependence widely occurs. In 1980–2005, the average growth rate of carbon dioxide emissions in developing and industrialized countries were, respectively, 285.8% and 53.6%, and world carbon dioxide emissions likely will increase by 49.6% in 2005–2020. Increased carbon dioxide emissions expand the demand for the ecosystem service of gas regulation (regulating service). Moreover, the average growth rate of fishery production (provisioning service) was 211.9% in developing countries and 17.6% in industrialized countries during 1980–2005.

The above analyses show that, in both the global scale and 152 countries, the human well-being represented by roundwood production, hydroelectricity generation and tourism investment were positively correlated with economic growth indicated by GDP during 1980–2005. Projected global roundwood production, hydroelectricity generation, tourism investment and GDP predict that these positive correlations are likely to exist still over the next 15 years. Dynamics of roundwood production, hydroelectricity generation, tourism investment and GDP in biodiversity hotspot and non-hotspot countries provide further evidence for these positive correlations. Moreover, higher growth rates of roundwood production, hydroelectricity generation and tourism investment illustrate greater demands for benefits from ecosystem services and biodiversity in hotspot countries than in non-hotspot countries.

Up to now, we have hardly made a comprehensive assessment of human well-being from ecosystem services and biodiversity yet, owing to the lacks of sufficient knowledge and appropriate technique [10], [17]. Roundwood production, hydroelectricity generation and tourism undoubtedly benefit from ecosystem services and their revenues are included into national accounting systems in the vast majority of the world's countries and regions. Accordingly, the value of relevant ecosystem services can be captured by the economic market. Comparisons of changes of PCRP, PCHG and PCTI in this study illustrate that the dependence of humans on cultural services has increased and will likely increase more rapidly than on regulating services, while the dependence on provisioning services has reduced and is projected to continue to reduce. The divergences in changes of PCRP, PCHG and PCTI are more pronounced in hotspot countries than in non-hotspot countries. Increased demands for regulating and cultural services (e.g. hydropower and tourism) can give humans an incentive to conserve ecosystems and biodiversity. Moreover, ecosystem degradation and biodiversity loss caused by the uses of regulating and cultural services are less than the use of provisioning service (e.g. roundwood production). Hence, the growths in dependence on regulating and cultural services are likely to promote the conservation of ecosystem services and biodiversity.

In this study, we explained that human well-beings yielded from ecosystem services were positively correlated with the stocks of ecosystem resources, by using roundwood production, hydroelectricity generation and tourism receipts as examples. Furthermore, ecological experiments, observations, and theoretical developments show that ecosystem properties depend greatly on biodiversity in terms of the functional characteristics of organisms present in the ecosystem and the distribution and abundance of those organisms over space and time [18]–[21]. These results show that humans can yield more well-being from ecosystem services and biodiversity that are better conserved. Moreover, we found that there is a close spatial correlation between the uses of ecosystem services and biodiversity hotspots. Effects of this spatial correlation on biodiversity are ambiguous. If humans are ignorant of societal dependence on natural ecosystems yet, this spatial correlation likely aggravates biodiversity loss. However, when the connections between ecosystems and human well-being are full understood, this spatial correlation will provide opportunities for biodiversity conservation.

The findings of our study have clearly demonstrated that economic growth has actually made humans more dependent upon ecosystem services and biodiversity than ever and this trend is most prevalent in developing countries with biodiversity hotspots that urgently need to build their conservation capacity [8], [22]. Evidently, we are asking more and more from natural ecosystems even as we reduce their capacity to meet our needs. As a consequence, our study suggests that *i)* it is crucial to consider the increase in human well-being yielded from ecosystem services along with economic growth when assessing the relationship between economic development and conservations of ecosystems and biodiversity; *ii)* a beneficial interaction between economic development and biodiversity conservation likely occurs [23]–[26], owing to the increase in dependence of humans on ecosystem services; and *iii)* the policies and implementations of both economic development and ecosystem/biodiversity conservation should be formulated and carried out in the context of the increased dependence of humans on ecosystem services along with economic development. Ecosystem service approaches to conservation offer a promising way to align conservation and production, simultaneously enhancing human well-being and protecting Earth's biodiversity and life-support systems [4], [27]–[32]. Achieving a sustainable world depends on a full understanding of the connections between ecosystems and human well-being and the drivers and responders to change [10]. Our study is a useful starting point for the understanding of the connections.

## Materials and Methods

152 countries involved in this study were chosen based on data availability. Roundwood production, hydroelectricity generation, tourism investment, GDP and population in the 152 countries accounted for 92%, 94.7%, 94.9%, 95.7% and 94% of the world ones during 1980–2005. The six representative hotspot countries were chosen from six major regions (Africa, Asia, Europe, North America, Oceania and South America) on the basis of regionally representative economic development and the spatial connection with biodiversity hotspots.

Biodiversity hotspot countries were identified according to Conservation International classification (http://www.biodiversityhotspots.org/xp/Hotspots/). The developing and industrialized countries among biodiversity hotspot countries were identified according to World Bank classification (http://www.worldbank.org/data/countryclass/classgroups.him).

Data and projections on roundwood production for the world and 152 countries over the period 1980–2020 are from World Resources Institute (http://earthtrends.wri.org) and Brown's literature [33]. Data and projections on GDP and hydroelectricity generation for the world and the 152 countries over the period 1980–2020 are from Energy Information Administration (http://www.eia.doe.gov/emeu/international/energy.html). Data and projections on tourism between 1988 and 2020 for the world and 152 countries are from The World Travel & Tourism Council (http://www.wttc.org/eng/Tourism_Research/Tourism_Satellite_Accounting_Tool/index.php). Populations between 1980 and 2020 for 152 countries, which were used in estimations of the changes in PCRP, PCHG and PCTI, are from United Nations Population Information Network (http://www.un.org/popin/data.html). Data on total forest area, water body area, grassland area and coastline length are from World Resources Institute (http://earthtrends.wri.org). The data on river systems are from World Resources Institute (http://earthtrends.wri.org). The data on carbon dioxide emissions were from Energy Information Administration (http://www.eia.doe.gov/emeu/international/energy.html), and the data on fishery production were from (http://earthtrends.wri.org).

Using Pearson's (*r*) correlation, we analyzed the correlation between the world annual GDP and the world annual roundwood production, the correlation between the world annual GDP and the world annual hydropower and the correlation between the world annual GDP and the world annual tourism investment, respectively, in 1980–2005. We also analyzed the correlation between total GDP and total roundwood production, the correlation between total GDP and total hydropower and the correlation between total GDP and total tourism investment in 152 countries in 1980–2005. Moreover, we analyzed the correlations between roundwood production, hydroelectricity generation and tourism receipts, respectively, and the stocks of ecosystem resources. Using Fisher's exact test, we tested for differences in the frequency of occurrence of hotspot and non-hotspot countries, and developing and industrialized hotspot countries in these categories, respectively.

## Supporting Information

Table S1 List of Hotspot and Non-Hotspot Countries related to this study.
(0.03 MB DOC)

Table S2 List of Industrialized and Developing Countries related to this study.
(0.03 MB DOC)
