# Supplementary material for: Increased Dependence of Humans on Ecosystem Services and Biodiversity
Source: PLoS One. 2010 Oct 1;5(10):e13113. doi: 10.1371/journal.pone.0013113 (PMC2948508; doi:10.1371/journal.pone.0013113)
Supplement: Table S1 — List of Hotspot and Non-Hotspot Countries related to this study. (0.03 MB DOC) [file pone.0013113.s001.doc]

# Table S1 List of Hotspot and Non-Hotspot Countries related to this study

**(1) Hotspot Countries**

Albania, Algeria, Argentina, Armenia, Australia, Azerbaijan, Bahamas, Bangladesh, Barbados, Belize, Benin, Bhutan, Bolivia, Bosnia and Herzegovina, Brazil, Cambodia, Cameroon, Cape Verde, Chile, China , Colombia, Comoros, Costa Rica, Croatia, Cuba, Dominican Rep, Ecuador, Egypt, El Salvador, Ethiopia, Fiji, France, Georgia, Ghana, Greece, Guatemala, Guinea, Haiti, Honduras, India, Indonesia, Iran Islamic Rep, Israel, Italy, Jamaica, Japan, Jordan, Kazakhstan, Kenya, Kyrgyzstan, Lao People's Dem Rep, Lebanon, Madagascar, Malawi, Malaysia, Mexico, Morocco, Mozambique, Namibia, Nepal, New Zealand, Nicaragua, Nigeria, Pakistan, Panama, Papua New Guinea, Paraguay, Peru, Philippines, Portugal, Puerto Rico, Russian Federation, Slovenia, South Africa, Spain, Sri Lanka, Sudan, Swaziland, Syrian Arab Rep, Tajikistan, Tanzania, Thailand, Togo, Tunisia, Turkey, Turkmenistan, Uganda, United States, Uzbekistan, Venezuela, Viet Nam, Zimbabwe

**(2) Non-Hotspot Countries**

Afghanistan, Angola, Austria, Belarus, Belgium, Botswana, Bulgaria, Burkina Faso, Burundi, Canada, Central African Rep, Congo (Brazzaville), Congo(Kinshasa), Czech Rep, Denmark, Equatorial Guinea, Estonia, Finland, French Guiana, Gabon, Germany, Guinea-Bissau, Guyana, Hungary, Iceland, Ireland, D. P. Rep of Korea, Rep of Korea, Kuwait, Latvia, Lesotho, Liechtenstein, Lithuania, Luxembourg, Macedonia (FYR), Maldives, Mali, Mauritania, Moldova, Rep, Mongolia, Netherlands, Niger, Norway, Poland, Qatar, Reunion, Romania, Rwanda, Senegal, Serbia and Montenegro, Slovakia, Suriname, Sweden, Switzerland, Ukraine, United Arab Emirates, United Kingdom, Uruguay, Western Sahara, Zambia
