# Supplementary material for: Increased Dependence of Humans on Ecosystem Services and Biodiversity
Source: PLoS One. 2010 Oct 1;5(10):e13113. doi: 10.1371/journal.pone.0013113 (PMC2948508; doi:10.1371/journal.pone.0013113)
Supplement: Table S2 — List of Industrialized and Developing Countries related to this study. (0.03 MB DOC) [file pone.0013113.s002.doc]

# Table S2 List of Industrialized and Developing Countries related to this study

1. **Developing Countries among Hotspot Countries**

Albania, Algeria, Argentina, Armenia, Azerbaijan, Bahamas, Bangladesh, Barbados, Belize, Benin, Bhutan, Bolivia, Bosnia and Herzegovina, Brazil, Cambodia, Cameroon, Cape Verde, Chile, China , Colombia, Comoros, Costa Rica, Croatia, Cuba, Dominican Rep, Ecuador, Egypt, El Salvador, Ethiopia, Fiji, Georgia, Ghana, Guatemala, Guinea, Haiti, Honduras, India, Indonesia, Iran Islamic Rep, Jamaica, Jordan, Kazakhstan, Kenya, Kyrgyzstan, Lao People's Dem Rep, Lebanon, Madagascar, Malawi, Malaysia, Mexico, Morocco, Mozambique, Namibia, Nepal, Nicaragua, Nigeria, Pakistan, Panama, Papua New Guinea, Paraguay, Peru, Philippines, Puerto Rico, Russian Federation, South Africa, Sri Lanka, Sudan, Swaziland, Syrian Arab Rep, Tajikistan, Tanzania, Thailand, Togo, Tunisia, Turkey, Turkmenistan, Uganda, Uzbekistan, Venezuela, Viet Nam, Zimbabwe

**(2) Industrialized Countries among Hotspot Countries**

Australia, France, Greece, Israel, Italy, Japan, New Zealand, Portugal, Slovenia, Spain, United States
